# Estimating the dispersal of the malaria vector *Anopheles farauti* through a natural ecosystem in north Queensland, Australia using mark release and recapture experiments

**DOI:** 10.1093/jme/tjaf143

**Published:** 2025-11-11

**Authors:** Weng K Chow, Robert D Cooper, Dan Pagendam, Brendan J Trewin, Nigel W Beebe

**Affiliations:** Australian Defence Force Malaria and Infectious Disease Institute, Vector Surveillance and Control, Gallipoli Barracks, Enoggera, Queensland, Australia; School of the Environment, The University of Queensland, St Lucia, Queensland, Australia; Australian Defence Force Malaria and Infectious Disease Institute, Vector Surveillance and Control, Gallipoli Barracks, Enoggera, Queensland, Australia; CSIRO, Health and Biosecurity, Dutton Park, Queensland, Australia; CSIRO, Health and Biosecurity, Dutton Park, Queensland, Australia; School of the Environment, The University of Queensland, St Lucia, Queensland, Australia

**Keywords:** Anopheles farauti, mark-release-recapture, dispersal, malaria vector

## Abstract

*Anopheles farauti* (Laveran, 1902) is a major malaria vector in the Southwest Pacific region that is showing behavioural resistance to indoor insecticide-based vector control. This study utilised fluorescent dust-based mark release recapture (MRR) experiments to quantify the dispersal characteristics of *An. farauti* through a non-village natural ecosystem in tropical north Queensland, Australia. Good quality adult mosquitoes were collected from the field using a Fan Box Traps (FBT), with initial parity dissections of this material showing a daily survival rate of 0.68 during the wet season and 0.75 during the dry season. We then performed 51 separate mosquito MRR releases (19,889 marked *An. farauti*) during the wet and dry seasons between 2015 and 2017. Mosquito recaptures utilised three collection systems: human landing catches (HLC), FBTs and a bespoke mosquito catch barrier system (MCB). Most of the 308 (1.55%) marked *An. farauti* were recaptured by the MCB (78%), while the rest (22%) were recaptured by either our CO_2_ baited FBT and HLC. The longest flight distance was 3.51 km and *An. farauti* was estimated to travel approximately 73 m per day with HLCs collecting approximately 14 times more mosquitoes than the CO_2_ baited FBT. This data provides novel insights into the movement and survival of *An. farauti* in a natural ecosystem, suggesting longer flight distances than documented and different survival rates between the wet and dry seasons. These parameters will be important for modelling pathogen transmission dynamics, potential mosquito gene drive spread, and contribute to future malaria control strategies in the Southwest Pacific.

## Introduction

Mosquito mark-release-recapture (MRR) studies have been used to try and quantify mosquito population dispersal patterns, which are important in understanding mosquito population biology and ecology (Silver 2008). Malaria vector MRR studies typically follows the female *Anopheles* mosquito movement through specific landscapes to better understand their behaviour and potential malaria transmission dynamics (Koffi et al. 2023). In general, mosquitoes disperse to mate, find a blood or sugar meal, rest, develop eggs, and oviposit. Adult mosquitoes also usually remain close to their larval site (Mackerras 1947) and studying flight behaviour is an important aspect in mosquito control and can be key to the management and modelling of disease spread (Service 1997).

Previous studies estimating the flight range of the Southwest malaria vector *Anopheles farauti* (Laveran, 1902) has reported considerable variation with estimates ranging 1.2–1.6 km on Espiritu Santo, New Hebrides (Vanuatu) using an indirect method of noting malaria cases in a definite area and then identifying the nearest larval habitat (Daggy 1945). Similarly, adult female *An. farauti* have also been captured 550 m offshore on a boat (Daggy 1945), and in Madang Province, Papua New Guinea (PNG), engorged female *An. farauti* flight ranges were estimated to be generally less than 50 m (Charlwood et al. 1985). In the Solomon islands, marked *An. farauti* were caught up to 700 m away from the release point (Hii 1988), and it has been suspected, that in areas of cleared forest, mosquitoes may track along the edge of the forest margin and fly up and down the road to reach their destinations (Charlwood and Wilkes 1981). Observations from other malaria vectors from Southeast Asia include the Indonesian vector *Anopheles barbirostris* (Van der Wulp, 1884), frequently flew up to 800 m within a single day of release (Davidson et al. 2019), and in the Philippines, *Anopheles minimus* (Theobald, 1901) and *Anopheles vagus* (Donitz, 1902) revealed flight distances ranged from 640 m to 1,470 m (Ejerctto and Urbino 1951). In Africa, efforts to characterize long distance migration of *Anopheles gambiae* (Giles, 1902), a tethered flight assay using a fixed tether and sound recordings to monitor flight, resulted in median flight time between 586 and 16,110 s, with flight aptitude peaking in the wet season (Faiman et al. 2020). Using similar flight mill systems (Rowley et al. 1968), flight distances of 9.0 km when sugar-fed and 10 km when blood-fed, while in starved females, the flight distance of below 3.0 km was recorded (Kaufmann and Briegel 2004). Early studies on *Anopheles* malaria vectors suggest they would fly below 10 m above ground level and its flight was key in determining its speed and direction rather than the wind (Snow and Wilkes 1972). A meta-analysis on the flight distance of mosquitoes that included 46 *Anopheles* species found an average flight distance based on MRR of 541.9 m with 1.6 km for *An. farauti*, and an average maximum distance for *Anopheles* being 3,490 m (Verdonschot and Besse-Lototskaya 2014). One recent study in the Sahel of Mali region of Africa shed new light on *Anopheles* mosquito dispersal where aerial sampling up to 290 m collected 10 different *Anopheles* species including *An. coluzzii* of which 80% of these mosquitoes were females and most had previously taken a blood meal, suggesting long distance dispersal may be common to these insects but rarely detected (Huestis et al. 2019). This study also simulated *Anopheles* flight ranges of up to 300 km (Huestis et al. 2019).

Studying mosquito flight behaviour enables a better understanding of a combination of natural behaviours observed in *An. farauti* mosquitoes and behaviours reported in other anophelines, such as host-seeking, migration, sugar-feeding, oviposition, swarming, and mating (Duffield et al. 2019). Such information may assist in understanding how the mosquito moves through different landscapes and contribute to our understanding of movement and potential mosquito control, including building parameters for modelling future gene drive systems for the targeted control of wild malaria-transmitting mosquito populations (Garrood et al. 2022).

The aim of this study is to estimate the flight range and dispersal characteristics of *An. farauti* through a non-village landscape of relatively native ecosystem at the Australian Defence Force’s Cowley Beach Training Area (CBTA) in north Queensland, Australia and this landscape represents a relatively natural ecosystem for this species. As MRR recapture rates can be low, we hypothesize that using large numbers of adult collected mosquitoes will provide insights into both the daily average and long-distance dispersal behaviour of *An. farauti* through a natural ecosystem in tropical north Queensland.

## Materials and Methods

### Study Site

The *An. farauti* MRR experiments were made at the CBTA (17°41′14.7″S 146°06′06.4″E) during both the wet (April) and Dry (October) seasons in 2015, 2016, and 2017. The CBTA, an Australian Defence Force (ADF) training area is situated approximately 40 km south of Innisfail. The training area comprises 5,081 ha of coastal lowland plains consisting of mixed open forest, melaleuca swamps, estuarine aquatics and rainforest to its North, West, and South and 8 km of beaches to the East (Buosi 2004). Tidal flats, cover approximately 37% of CBTA and consist mainly of regularly inundated areas with mangroves and tidal creeks (Buosi 2004).

The climate type for the area is monsoonal with a distinct wet (November to April) and dry (May to October) season. There is seasonality in the temperature with May to October being the cooler months and November to April the hotter months. Innisfail receives a mean annual rainfall of 3,547 mm per year and a high mean relative humidity of 75%–80% that is constant throughout the year (Australian Bureau of Meteorology 2022).

### Collecting Adult Female *Anopheles farauti*

**Fig. 1.**
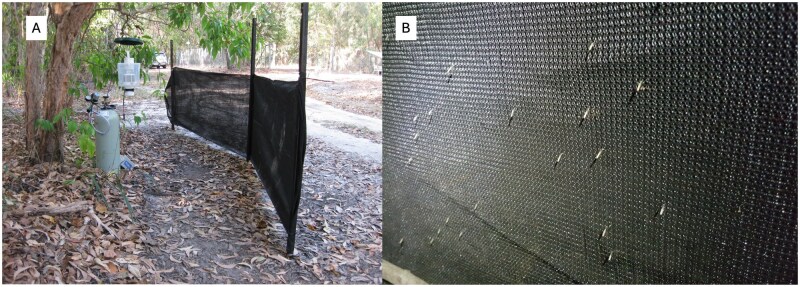
(A) The mosquito catch barrier system combines a fan box trap that uses CO_2_ as an attractant with a barrier screen. (B) *Anopheles farauti* can be easily seen resting on the barrier screen of the mosquito catch barrier system (MCB).

**Fig. 2.**
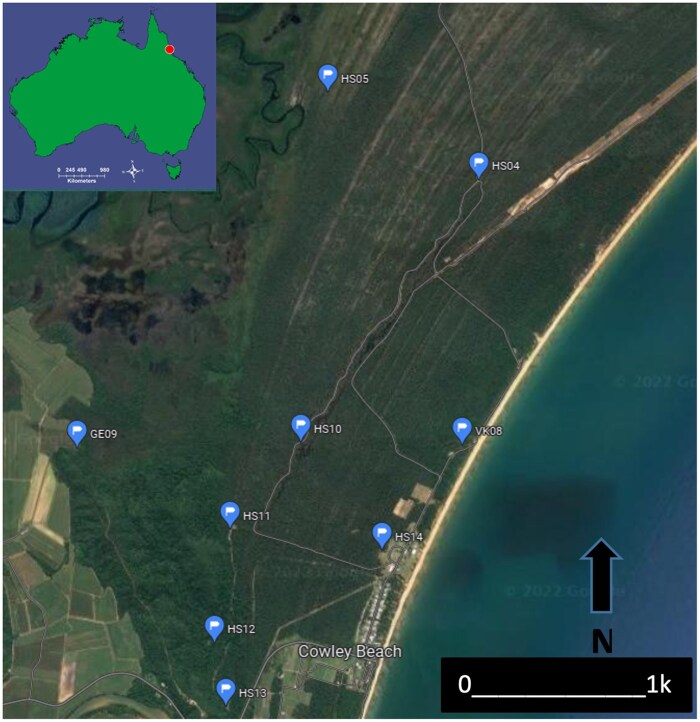
Release and recapture locations at Cowley Beach Training Area, north Queensland, Australia represents a relatively natural landscape of mixed open forest, melaleuca swamps, estuarine aquatics, and rainforest. The fluorescently dusted mosquitoes were released from sites HS11 and HS14.

Adult female *An. farauti* mosquitoes were collected for marking at site HS11 and HS13 between the times of 1800–2200 h using three methods: (i) a novel mosquito catch barrier system (MCB) (Fig. 1A), (ii) CO_2_ baited Fan Box Traps (FBT) (Chow et al. 2024), and (iii) human landing catches (HLC) (Fig. 2 and Table 1). Mosquito collections by FBT and HLC were performed as described (Chow et al. 2024) and (Chow et al. 2023), respectively. The MCB were made of a 70% black ultraviolet (UV), high-density polyethylene (HDPE) sunshade cloth (Model#: 314453, Blackwoods, QLD, AUS). The construction design was a four-metre straight length by one-metre-high single strip of shade cloth, attached to star pickets with black zip ties and positioned one metre away from an associated FBT (Fig. 1A). The FBT was dual purposed as a live mosquito catch system and attractant for mosquitoes onto the MCB. The FBT CO_2_ gas release rate was set at 500 ml/min. The MCB utilised the FBT down draft trap design to concentrate the CO_2_ plume against the inner side of the MCB. This method was effective in attracting the mosquitoes to rest on the MCB (Fig. 1B). The black shade cloth enabled quick identification of adult *An. farauti* for collection by mouth aspiration into a tube. The MCB was sampled each quarter hour during the four-hour sampling period. Unanesthetized female *An. farauti* were counted and separated into quantities of 50 per wax lined cup Ripple Wall Coffee Cups (227 ml, 75 mm diameter, and 90 mm height) and retained in the cups with mosquito netting until ready for marking.

**Table 1.** Mosquito trap and release sites

Serial	Collection site	Latitude	Longitude	Remarks
**1**	GE09	S17°40′54.5″	E146°05′28.3″	Trap site
**2**	HS04	S17°39′48.1″	E146°07′08.6″	Trap site
**3**	HS05	S17°39′26.5″	E146°06′30.7″	Trap site
**4**	HS10	S17°40′53.2″	E146°06′24.1″	Trap site
**5**	HS11	S17°41′14.7″	E146°06′06.4″	Trap site and Release site
**6**	HS12	S17°41′42.7″	E146°06′02.4″	Trap site
**7**	HS13	S17°41′58.5″	E146°06′05.3″	Trap site
**8**	HS14	S17°41′19.9″	E146°06′44.2″	Release site
**9**	VK08	S17°40′53.8″	E146°07′04.4″	Trap site

### Mark, Release, and Recapture of Adult Female *Anopheles farauti*

#### Mark

The maximum of 50 *An. farauti* mosquitoes held in each wax cup were dusted with a small amount (two rice grain worth) of fluorescence coloured dust (Glow Paint Industries, QLD, Australia) by using a scalpel blade (Model#:0507 size 21, BSN Medical Pty Ltd, VIC, Australia) and smearing it across the top of the mosquito netting. This created a powdery plume inside the cup, which lightly covered the entire mosquito.

#### Release

**Fig. 3.**
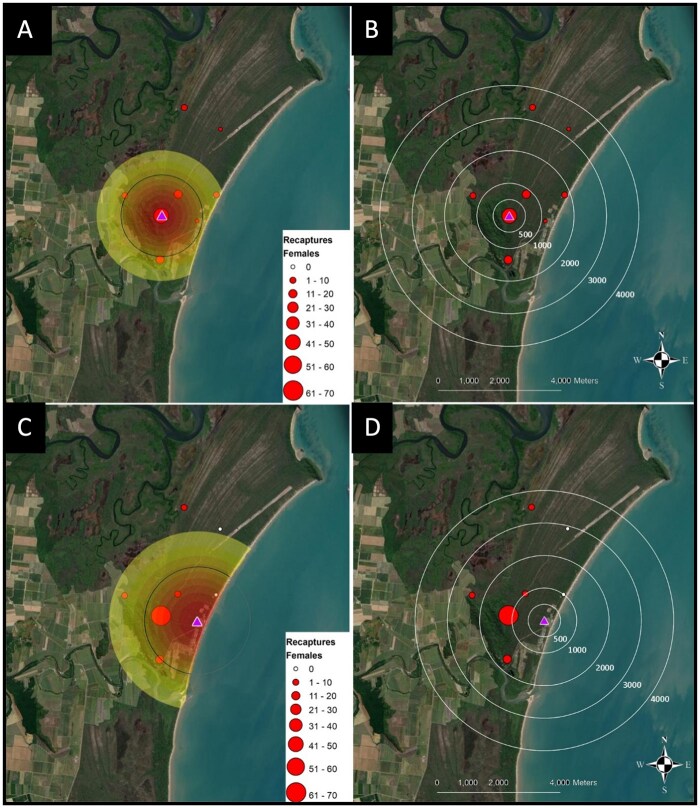
Total dusted *An. farauti* adults captures after releases at site HS11 (A, B) and at site HS14 (C, D). Size and colour of circles indicate the total *An. farauti* adults captured. Release points are indicated by purple triangle. Estimates of *An. farauti* movement using the STEIK framework (A, C) where concentric circles from release points are density estimates of dusted *An. farauti*. Black lines represent mean distance travelled over time.

In total, 51 separate cohorts of unfed wild caught adult female mosquitoes were released from two different locations (site HS11 and HS14) (Figs 2 and 3). Release sites were selected based on the open landscape with the sea located to the east, while to the west was native mixed opened forest and rainforest. The releases from each location were distinguished using two different colour of fluorescent dust—green and orange (Model#: fluroesce-01, Glow Paint Industries, QLD, Australia). Each cohort of mosquitoes were released at 2300 h, and of the 51 releases performed, 22 were carried out in the wet seasons (April) and 29 occurred in the dry seasons (October).

#### Recapture

Adult recaptures were undertaken using two collection methods: (i) FBT and (ii) HLC. The FBTs were deployed in the surrounding landscape of CBTA at eight locations (Fig. 2 and Table 1) and set at 1800–0600 h each night at various distances (500–1,000 m apart) from the mosquito release sites. The FBT locations were set at various types of different landscapes and distances to observe the ability of adult female *An. farauti* mosquitoes to fly through or above the different environments that exist in CBTA. The traps were collected daily. The HLC was only employed at site HS11 using one individual from 1800 h until midnight and then a second individual from midnight to 0600 h. All *An. farauti* mosquitoes captured were visually checked under a stereomicroscope with a UV black light torch to determine if it has been dusted with a fluorescent dust and the site of collection, marking colour, date and number recaptured were recorded. The distance from release point to recapture point was calculated using a 1:50,000 scale topographic map of CBTA.

### Longevity of a North Queensland *Anopheles farauti* Population

Ovaries of the collected *An. farauti* were dissected in physiological saline and allowed to air dry. Ovaries were examined under a ×100 compound microscope and if the tracheoles ended in skeins, it was nulliparous and if the skein have been extended out then it was parous (Detinova 1962). The wet and dry season survival estimates were calculated from the proportion parous at CBTA via the methods of (MacDonald 1952, Detinova 1962) where the proportion parous (P) is used to determine the survival through one day (*p*) as ^*x*^√*P*; where *x* is the length of the gonotrophic cycle. An estimated gonotrophic cycle of 2.5 days was used from other studies from the SWP region (Charlwood et al. 1985, Hii 1988, Birley and Charlwood 1989, Bugoro et al. 2011a, Russell et al. 2016), as it was too difficult to obtain an accurate gonotrophic result for *An. farauti* at CBTA.

### Species Identification and Population Size Estimates

All live *Anopheles* collected were initially identified by adult morphology to the *An. farauti* complex by the naked eye (Marks 1982). As two other isomorphic species—*Anopheles hinesorum* (Schmidt et al. 2001, formally *An. farauti* 2) and *Anopheles torresiensis* (Schmidt et al. 2001, formally *An. farauti* 3) are can also occur in north Queensland coastal region (Van Den Hurk et al. 2000), subsets of anophelines from each collection period and recaptured dusted mosquitoes were preserved in 70%–100% ethanol for transport back to the laboratory where *Anopheles* specimens underwent individual salt-extractions to obtain genomic DNA (Beebe et al. 2001) and species identified by diagnostic PCR based on a *Msp*I restriction digest of the Internal Transcribed Spacer region 2 (Beebe and Saul 1995).

Estimations of population size for the wet and dry seasons in 2015, 2016 and 2017 were calculated using the Lincoln–Peterson Index (Southwood and Henderson 2009) assuming all released and recaptured were *An. farauti*.

### Determination of Biological Parameters, Statistical Analysis and Dispersal Kernel Framework

Records of the numbers of dusted female mosquitoes recaptured for both the FBT and HLC collections were used to fit a simple dispersion model in two-dimensional space (see Supplementary File S1 for data summary). The dispersion model assumed that mosquito movement can be modelled according to the laws of diffusion, whereby the post-release density of mosquitoes follows a bivariate Gaussian distribution (in 2D-space) that evolves through time (Trewin et al. 2020). The approach is referred to as a spatially and temporally evolving isotropic kernel (STEIK) framework. We allowed for the dispersion to also have a prevailing drift direction (uniform across the study area) that could arise because of prevailing winds or other environmental gradients that might result in a preferential movement direction. Our diffusion model also accounted for mosquito death, so that the likelihood of recapturing each released mosquito diminished with time since release.

Mathematically, the model for the mosquito density (in time and space) dusted with a particular colour, was


(1) λ(t,x,y)={0, if t<t1∑r=1R(t)nre-μ(t- tr)e-1σ2tr{(x-(xr+ μxt))2+(y-(yr+ μyt))2}2πσ2tr, if t≥ t1,

where t denotes a particular point in time, measured in days following one or more releases; the pair (x,y) denotes the Cartesian coordinates in metres of a point in space; R(t) is the number of releases undertaken prior to time t; $t_{r}$ is the time of the *r*th release; $\left(x_{r},y_{r}\right)$ is the location of the *r*th release; $n_{r}$ is the number of dusted female mosquitoes released at the *r*th release; $\mu_{x}$ and $\mu_{y}$ are the prevailing drifts in the *x* and *y* directions, respectively (measured in metres per day); and $\sigma^{2}$ is the diffusivity of the mosquito dispersion.

Several data models were considered for coupling the spatio-termporally evolving mosquito density (as a result of dispersion) to the observed counts. In the simplest data model, the counts observed in the field were modelled as a Poisson process whose intensity function was proportional to the modelled density of the mosquitoes shown in (1). We also considered negative-binomial, a generalized Poisson, and zero-inflated (Z.I.) versions of the Poisson, binomial, and generalized Poisson as candidate data models. To relate the trap counts back to our dispersion model, we used two strictly positive coefficients ($K_{\mathrm{FBT}}$ and $K_{\mathrm{HLC}}$) that act as trapping efficiencies: converting the mosquito densities into the expected number of mosquitoes caught for each trap type. An additional parameter, α, provided a mechanism by which the expected number caught in the wet season can be higher or lower than in the dry season. Our alternative data models for the trapping counts are written as


(2) $$Y_{T}(t_{a},\;t_{b},\;x,y_{T})∼\text{Poisson}(K_{T}\mu (t_{a},\;t_{b},\;x,y_{T})),$$

(3) $$Y_{T}(t_{a},\;t_{b},\;x,y_{T})∼Z.I.-\text{Poisson}(\pi_{T},K_{T}\mu (t_{a},\;t_{b},\;x,\;y_{T})),\;$$

(4) $$Y_{T}(t_{a},\;t_{b},\;x,\;y_{T})∼\text{Neg}.-\text{Bin}.(K_{T}\mu (t_{a},\;t_{b},\;x,y_{T}),\;n),$$

(5) $$Y_{T}(t_{a},\;t_{b},\;x,\;y_{T})∼Z.I.-\text{Neg}.-\text{Bin}.({\pi_{T},K}_{T}\mu (t_{a},\;t_{b},\;x,\;y_{T}),\;n),\;$$

(6) $$Y_{T}(t_{a},\;t_{b},\;x,\;y_{T})∼\text{Gen}.-\text{Poisson}(K_{T}\mu (t_{a},\;t_{b},\;x,y_{T}),\;\varphi ),\;$$

(7) $$Y_{T}(t_{a},\;t_{b},\;x,\;y_{T})∼Z.I.-\text{Gen}.-\text{Poisson}(\pi_{T},\;K_{T}\mu (t_{a},\;t_{b},\;x,\;y_{T}),\;\varphi ),\;$$

where


$$\mu (t_{a},\;t_{b},\;x,\;y)=K_{T}(1+\;\alpha I\{\text{wet}\})\int_{t_{a}}^{t_{b}}\lambda (t,x,y)dt.$$

In the above equation, T∈{FBT, HLC} denotes the trap type, ${[t}_{a},\;t_{b}]$ defines a time interval in decimal days over which trapping was conducted; (x,y) denotes the Cartesian coordinates in metres of the trap; $K_{T}$ denotes the trapping efficiency for trap type T; I{wet} is an indicator variable that takes the value 1 if the data was captured in the wet season and zero otherwise; and α is a parameter that can increase (positive value) or decrease (negative value) the expected trap catch for the wet season from a dry season baseline. The probability mass functions for the Poisson distribution is


$$p_{P}(y;\mu )=\frac{\mu^{y}e^{-\mu }}{y!};$$

with μ being the mean of the distribution which is taken to be equal to $\mu (t_{a},\;t_{b},\;x,\;y)$. For the negative-binomial distribution we have


$$p_{NB}(y;\mu ,\varphi )=(\begin{array}{c} y+\varphi -1\\ y \end{array}){\left(\frac{\mu }{\mu +\varphi }\right)}^{y}{\left(\frac{\phi }{\mu +\varphi }\right)}^{\varphi },$$

with μ being the mean of the distribution which is taken to be equal to $\mu (t_{a},\;t_{b},\;x,\;y)$ and the variance is equal to φμ. For the generalized Poisson distribution (Consul and Jain 1973, Zamani and Ismail 2012) we have


$$p_{GP}(y;\mu ,\varphi )=\frac{\mu {\left(\mu +\varphi y\right)}^{y-1}}{{\left(1+\varphi \right)}^{y}y!}\text{exp}\;(-\frac{\mu +\varphi y}{1+\varphi }),$$

with μ being the mean of the distribution which is taken to be equal to $\mu (t_{a},\;t_{b},\;x,\;y)$ and variance equal to ${\left(1+\varphi \right)}^{2}\mu$. The zero-inflated extensions of these probability mass functions can be written as


pZI-X(yT;πT, ·)= {πT+(1-πT)pX(0; ·) (yT=0),(1-πT)pX(yT; ·) (yT>0),

where  X∈{P, NB, GP}. The use of a dot in the brackets of a function is used to mean any other arguments that are not explicitly present.

One complication with the model is that at the instance that a mosquito cohort is released at a location, the density, λ(t,x,y), is infinite. To avoid associated numerical issues, we assume that at site HS11 (which was both a trapping and release site), the trap was located at 50 m from the release point.

To also estimate movement for *An. farauti* we applied the spatially and temporally evolving isotropic kernel (STEIK) framework developed by Trewin et al. (2020). Dispersal kernels on maps based on recaptures in FBTs and individual dusted mosquito release sites were parameterized and mean distance travelled (MDT) was estimated using an isotropic Gaussian diffusion model (Trewin et al. 2020). The MDT estimates were estimated to reflect variations across season and release site via Monte Carlo simulation. One million lifetimes ${(t}_{1},\ldots ,t_{1,000,000})$ were randomly sampled from the adult female *An. farauti* lifetime distributions, where average life expectancy (ALE) was calculated from dry and wet season survival estimates (Niebylski and Craig 1994). For each sampled lifetime ($t_{i}$) a distance travelled, $d_{i}$, was sampled from the χ-distribution above, but where t was set equal to $t_{i}$. For MDT from HS14 and HS11 we applied the dry and wet season ALE, respectively. The resulting values for the distances travelled in a lifetime, $d_{1},\ldots ,d_{1,000,000}$, were then used to compute the MDT from each release site (Trewin et al. 2020). Spatial kernels were plotted in R using a density function with a mean of 0 and a variation equal to the standard deviation estimated from the STEIK framework for each release site and then plotted as an image with a circle equivalent to the MDT. R kernel images were overlaid on maps in photoshop where the kernel MDT circle was equivalent to a buffer ring of the same distance from release sites.

### Ethics

For this research, ethical approval was granted from the Australian Defence Human Research Ethics Committee—Protocol number: 774-14 (10 April 2015), Australian Defence Force Malaria and Infectious Disease Institute (ADFMIDI) Biosafety committee and ADFMIDI Work Health and Safety committee for conducting human landing catch on this study.

## Results

During the MRR study, 19,889 *An. farauti* mosquitoes were dusted and released from 51 cohorts ranging from 44 to 1,731 mosquitoes per release (Supplementary File S1). The release and recapture rates for the wet and dry season experiments are shown in Table 2 along with the Lincoln–Peterson estimates for abundance for each season. A summary of the total recaptures for each trap shown in Supplementary Table S1 with a total of 308 dusted *An. farauti* recaptured (1.55%). Recapture rates differed between 1.25% and 1.98% from the HS11/HS14 release sites and 1.08%–1.77% from wet/dry season, respectively. Subsets of *An. farauti* mosquitoes from each collection period (wet/dry seasons) and recaptured dusted mosquitoes were confirmed as *An. farauti s.s.* by molecular analysis (*n* = 250), with no other cryptic species identified. Assuming all captured and recaptured mosquitoes were *An. farauti*, the abundance estimates based on the Lincoln-Peterson estimation in the wet and dry seasons are displayed in Table 2.

**Table 2.** *Anopheles farauti* abundance estimates in the wet and dry seasons

Year-season	Marked & released	Total collected	Marked collected	Population size
**2015-wet**	3,346	16,675	39	1,430,629
**2015-dry**	1,110	17,425	14	1,381,553
**2016-wet**	2,374	17,051	19	2,130,478
**2016-dry**	5,422	16,997	119	774,434
**2017-wet**	752	4,287	13	247,986
**2017-dry**	6,885	2,599	105	170,420

The furthest flight recapture of three *An. farauti* females was recorded at a trap site 3.51 km from their release point (HS05). There also appeared to be clustering at the collection site HS11, where 64.9% (200/308) of the recaptures were made.

### Longevity of Adult Female *Anopheles farauti* Populations in North Queensland

Parity dissections were used to estimate the daily survival rates (MacDonald 1952, Davidson 1954, Detinova 1962) and were performed on 1,361 and 1,801 adult female *An. farauti* specimens collected in the wet and dry seasons, respectively. The overall parity was found to be 0.39 and 0.49 in the wet and dry seasons, respectively (Table 3), with the daily survival rate (*p*) of the CBTA *An. farauti* population to be 0.68 during the wet season and 0.75 during the dry season.

**Table 3.** The proportion of parous mosquitoes within the *Anopheles farauti* population in Cowley Beach Training Area calculated from the wet and dry season collections

Year	Season	
	Wet	Dry
**2015**	0.38	0.52
**2016**	0.38	0.53
**2017**	0.39	0.46
**Overall parity**	0.39	0.49
**Daily survival (*p*)**	0.68	0.75
**Total mosquitoes (*n*)**	1,361	1,801

### Adult Female *Anopheles farauti* MRR Modelling

The models outlined above were fitted using maximum-likelihood with optimization of the log-likelihood carried out using the BFGS method provided in the “optim” function within R (R-Core-Team 2017). The integral within the expression for $\mu (t_{a},\;t_{b},\;x,\;y)$ was evaluated numerically by discretizing time into five-minute intervals and employing a trapezoidal approximation. The Bayesian Information Criterion (Schwarz 1978) for each model was computed (Table 4), with the model having the lowest value selected as most appropriate. On this basis, the Generalised-Poisson model was considered most appropriate for the data. Once the maximum likelihood estimates of the model parameters were found, 95% confidence intervals for the model parameters were obtained using profile-likelihood. The large sigma parameter (dispersal parameter) showed combined green and orange coloured release estimates of 1,064 m—1,433 m (95% C.I.; Table 5). Two other separate analyses for green and orange dusted mosquito releases excluding the HLC parameter (to evaluate flight range difference using different dusting colours) showed 786 m–1,149 m (95% C.I., green dusted releases, Supplementary Table S2, Fig. 3B) and 1,067 m–1,562 m (95% C.I., orange dusted releases, (Supplementary Table S3 and Fig. 3D). The STEIK kernel MDT estimated for each release site was 1,263.9 m (wet season survival 0.68) and 1,650.2 m (dry season survival 0.75) from HS11 and HS14, respectively (Fig. 3A and C). A very small death rate (beta parameter) was also observed. No significant effect of wet season (alpha parameter) on trapping rate for the green dusted mosquitoes and a significant, reduced trapping rate (alpha parameter) was observed in the wet season for the orange dusted mosquitoes (Supplementary Tables S2 and S3).

**Table 4.** Log-likelihoods and Bayesian information criterion (BIC) for different count data models

Model	Log-likelihood	Number of parameters	Number of observations	Bayesian information criterion
**Poisson**	−479.83	7	938	1,007.57
**Zero-inflated Poisson**	−447.27	9	938	956.13
**Generalised Poisson**	−443.89	8	938	942.53
**Negative Binomial**	−449.36	8	938	953.47
**Zero-inflated Negative Binomial**	−445.18	10	938	958.79
**Zero-inflated Generalised Poisson**	−438.99	10	938	946.42

**Table 5.** Parameter estimates and 95% confidence intervals obtained for the model of mosquito movement using the Generalised-Poisson model for trap counts

Parameter	Maximum likelihood estimate	Lower 95% C.I.	Upper 95% C.I.
**Movement drift in *x*-direction (** $\mu_{x}$ **)**	−43.199	−293.70	78.700
**Movement drift in *y*-direction (** $\mu_{y}$ **)**	−58.836	−273.84	66.364
**Movement dispersion (** σ **)**	1,229.2	1,064.9	1,433.2
**Death rate (** β **)**	0	0	9.0088E-3
**Wet season effect (** α **)**	−0.30927	−0.59627	0.084359
**Box fan trap catch rate modifier (** $K_{\mathrm{BFT}}$ **)**	7,223.8	5,561.5	9,424.1
**Human landing trap catch rate modifier (** $K_{\mathrm{HLT}}$ **)**	104,142	61,176	159,533
**Dispersion parameter of Generalised-Poisson trap counts (** φ **)**	0.39048	0.20849	0.65582

## Discussion

The *An. farauti* population at CBTA in Australia’s tropical north Queensland exists in a relatively natural coastal ecosystem and has never undergone malaria vector control including indoor insecticide-based selection pressure such as indoor residue spraying, or insecticide treated nets. The daily survival rate of this population was estimated at 0.68 during the wet season and 0.75 during the dry season, similar to another Australian study with a survival rate of 0.75 in Casuarina, Darwin (Russell 1987). The higher daily survival rate in the dry may be due to high and heavy rainfall in the wet season—a seasonal survival pattern also observed with the Amazon malaria vector *An. darlingi* and attributed to rainfall (de Barros et al. 2011). However, this apparently lower daily survival rate at CBTA was not observed with studies in equatorial northern PNG coastal village of Bilbil, Madang (Afifi et al. 1980), Wewak, PNG (0.88) (Peters and Standfast 1960), D’Entrecasteaux Islands (0.85) (Spencer 1965), or *An. farauti* in the Solomon Islands (Bugoro et al. 2014) where parous rates of 0.54–0.58 suggests daily survival rates of 0.78–0.80 using the methods of Davidson (1954). Additionally, this CBTA *An. farauti* population daily survival rate is also lower than studies on *An. gambiae* and *An. arabiensis* in Tanzania (0.80) (Charlwood et al. 2018). However, another study in the more equatorial Madang Province, PNG, recorded daily survival estimates for *An. farauti* ranged between 0.32 to 0.64 in the wet season and 0.49 to 0.73 in the dry season (Charlwood et al. 1985). It appears, variations in these estimated daily survival rates were likely attributed to authors using different gonotrophic cycles rates and potential under sampling at collection sites and seasons. This discrepancy may be also due to differential dispersal or concentrations of different mosquito age classes e.g. younger age classes more common nearer to breeding sites (Charlwood et al. 1985) and inclement weather events during collections. There was little variation in temperature and humidity between the studies with mean temperatures ranging from 26 to 28 °C and mean humidity ranging from 74% to 85% (Peters 1960, Spencer 1965, Charlwood et al. 1985, Bugoro et al. 2011b). The gonotrophic cycle is also considered constant in tropical environments, but because it’s dependant on temperature, it will vary in temperate areas, therefore, accurate estimations on daily survival rates may become impossible (Gillies 1974).

The major aim of this work was to estimate the movement of *An. farauti* through a natural ecosystem in tropical Queensland using MRR experiments. We released 19,889 fluorescently dusted unfed *An. farauti* female mosquitoes and 308 (1.55%) were recaptured using a combination with trapping methods including MCB, FBT, and HLC. The daily flight range estimation was 73 m/day, and we recaptured three females at the furthest trap, which was 3.51 km from the release site. This trap distance is the longest flight range observed for *An. farauti* adult females to date, however, limitations on the availability of traps, access to trap sites and the influence of being dusted (mosquitoes are often observed grooming the coloured dust off their bodies when released), suggest they could likely fly further. The collection sites HS11, showed highest recapture rate (Fig. 3). These higher recapture rates may be explained by the closer proximity of the trap to the release points as these unfed mosquitoes released at site HS11, would move into the adjacent rainforest to rest and seek harbourage before seeking a blood meal detecting the nearest CO_2_ plume cues, although some form of homing cannot be discounted.

**Fig. 4.**
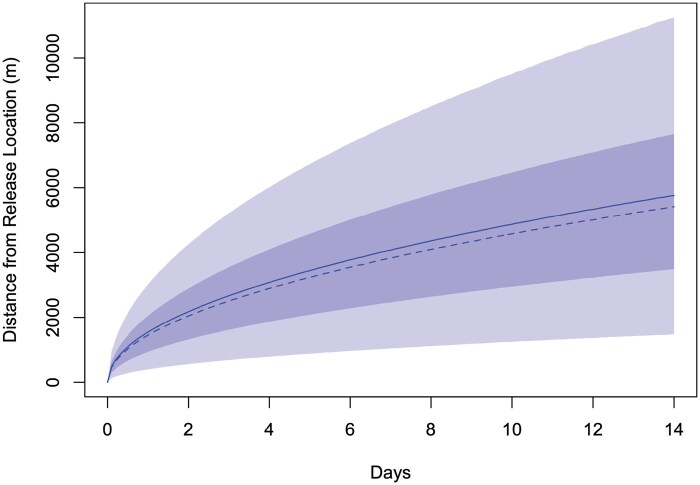
Plot of the absolute distance travelled from the release location for female An. farauti mosquitoes. The solid line shows the mean; the dashed line shows the median; light shading shows the 90% confidence interval; and dark shading shows the 50% confidence interval.

Estimates of dispersal parameters from the STEIK framework with a Generalised-Poisson data model provide insights for combined and individual green and orange dusted mosquito releases. First, all three Generalised-Poisson model results portrayed a consistent picture of mosquito movement dynamics. For the analysis using all data (HLC and FBT; both colour releases) in a single model, there was statistically significant evidence of preferential movement in a southwest direction at a rate of approximately 73 m per day (Table 3). However, when the analyses were split by colour and HLC data was removed (Supplementary Tables S2 and S3), drift in both the west and south directions was not statistically significant, providing a lack of strong evidence of a preferential movement (drift) direction (the 95% confidence intervals contained zero). Second, the dispersion parameter σ provided valuable information regarding how far mosquitoes had dispersed via random-walk dynamics in addition to the preferential drift. Results summarized in Fig. 4 and Supplementary Fig. S1 shows the expected absolute distance that mosquitoes would have travelled from the release site as a function of days since release, using the maximum likelihood estimate of σ, and assuming no drift ($\mu_{x}$ and $\mu_{y}$ set equal to zero) with the same outcome for the different coloured markers. The large sigma parameter (dispersal parameter) showed similar dispersal ranges (95% C.I.) for both combined and individual coloured dusted mosquito releases, which indicates that there is no difference to mosquito dispersal flight ranges when using different coloured dust on mosquitoes (Table 3 and Supplementary Tables S2 and S3). Third, in all models, the death-rate parameter, β, was estimated to be very small (numerically equivalent to zero). As the maximum likelihood estimate of this parameter may lie on the boundary of the parameter space, the confidence intervals obtained using profile-likelihood may be inadequate. Fourth, the confidence interval for α contains zero, therefore, there is not strong evidence to suggest that counts differ greatly between the wet and dry seasons. Finally, the parameters $K_{\mathrm{FBT}}$ and $K_{\mathrm{HLC}}$ allow us to quantify the density-independent rate at which FBT and HLC capture female mosquitoes (Table 3). Interestingly, $K_{\mathrm{HLC}}$ is over 14 times larger than $K_{\mathrm{FBT}}$ which indicates that a HLC will catch approximately 14 times more mosquitoes than a FBT, when exposed to the same density of mosquitoes for the same period of time. These results provide evidence that HLC remains important for estimating human exposure to mosquitoes and as part of estimating the entomological inoculation rates, until we develop a comparable alternative mosquito trap solution. Nonetheless, HLC methods have important limitations that restrict their use in areas exposed to antimalarial drug-resistant parasites or active arbovirus transmission.

There are three assumptions underpinning our mathematical model and that might be regarded as a limitation of the analyses. First, we assume a simple and time-homogeneous model of dispersion of marked mosquitoes with each mosquito moving according to a simple random walk with drift in one direction. It is likely that mosquito movement is driven by many sensory cues, but as we have no information regarding these, we cannot include these as processes in the movement model. Our second assumption is that traps of a certain type will all operate with a particular trap efficiency. This may not be the case, with some traps potentially placed in locations that are less effective or showing variability in their effectiveness to lure. Finally, our model assumes that the rate at which mosquitoes are caught in a trap is linearly related to their density (as dictated by the movement model). This may be reasonable if mosquitoes all behave independently of one another, but could also be a limitation where mosquito behaviour (and catchability) actually changes as a function of their density.

The flight range and dispersal characteristics of adult female *An. farauti* presented in this study may also be used for modelling potential population modification strategy such as genetically engineered gene drive mosquitoes as tools to decrease the burden of malaria by either population suppression or by driving pathogen refractory traits through disease-transmitting mosquitoes (Nolan 2021, Garrood et al. 2022, Geci et al. 2022, Carballar-Lejarazú et al. 2023). This study suggests the flight distance for *An. farauti* is larger than that found by other studies in the Southwest Pacific (SWP) (Daggy 1945, Charlwood et al. 1985, Hii 1988). Although, other flight range studies on malaria vector species in Southeast Asia have showed similar results to *An. farauti* (Ejerctto and Urbino 1951, Davidson et al. 2019). All these studies likely underestimate mosquito movement as we are deriving dispersal estimates often less that 2% of released mosquitoes, so the use of MRRs for estimating mosquito movement should be viewed with some caution due to the limitations of the experimental procedure. When compared to some different African *Anopheles* species studies that have either estimate tethered mosquito’s flight hours (Kaufmann and Briegel 2004, Faiman et al. 2020), or estimated long distance dispersal through detecting mosquitoes travelling at elevation (Huestis et al. 2019), MRRs have limitations but their utility for detecting dispersal behaviors over small spatial scales provides valuable insights into mosquito behaviour. As we counted all recaptured *An. farauti* mosquitoes with dust markings, there is the possibility of dust transfer to an undusted *An. farauti* in the collection containers. As the trapping containers contained mixtures of mosquito species, we considered this possibility to be unlikely.

The longest detectable flight for an adult female *An. farauti* in CBTA was 3.51 km (the maximum measurable distance in this study), which is close to the average maximum distance across 46 species of *Anopheles* of 3,490 m, (Verdonschot and Besse-Lototskaya 2014). As the coastally restricted *An. farauti* has essentially a one-dimensional (linear) distribution, a larger trapping configuration may suffice to generate longer distance estimates. Alternatively, genetic tools that examine close kin relationships of mother-offspring, father-offspring, full-sibling and half-sibling pairs could also be performed either through MRRs (Sharma et al. 2022), or by using spatial genetics (Filipović et al. 2020). Understanding the causative factors (environment, season, location) and underlying differences in flight activity would have real implications for addressing the alternative mosquito control requirements of these vector species such as genetic modification, with most requiring initial mathematical modelling to simulate the spread of a trait/modification (Sánchez et al. 2020, Beeton et al. 2022).

In summary, the daily survival rate and dispersal estimates for female *An. farauti* population have been presented in a wild population naive to antimalaria insecticidal vector control pressure and this study occurred in a relatively natural ecosystem for *An. farauti* (non-village setting) in tropical north Queensland, Australia. These survival and dispersal parameters will also contribute to modelling mosquito movement for future activities including the exploration of the potential a gene-drive malaria refractory *An. farauti* as a future malaria control tool.

## Supplementary Material

tjaf143_Supplementary_Data
