# Supplementary material for: Estimating the dispersal of the malaria vector Anopheles farauti through a natural ecosystem in north Queensland, Australia using mark release and recapture experiments
Source: J Med Entomol. 2025 Nov 11;63(1):tjaf143. doi: 10.1093/jme/tjaf143 (PMC13055874; doi:10.1093/jme/tjaf143)
Supplement: tjaf143_Supplementary_Data [file tjaf143_supplementary_data.zip › Fig_S1-Figure_legend.docx]

**Fig. S1.** Plot of the absolute distance travelled from the release location (site HS11 – Green and site HS14 – Orange) for female *An. farauti* mosquitoes. Solid blue line shows the mean; dashed line shows the median; light blue band shows the 90% confidence interval; and dark blue band shows the 50% confidence interval.
