# Supplementary material for: Estimating the dispersal of the malaria vector Anopheles farauti through a natural ecosystem in north Queensland, Australia using mark release and recapture experiments
Source: J Med Entomol. 2025 Nov 11;63(1):tjaf143. doi: 10.1093/jme/tjaf143 (PMC13055874; doi:10.1093/jme/tjaf143)
Supplement: tjaf143_Supplementary_Data [file tjaf143_supplementary_data.zip › Tables S1-S3.docx]

**Table S1.** Summary of An. farauti, marked, released and recaptured at Cowley Beach Training Area trap sites.

| **Release**  **Site/#** | **HS04** | **HS05** | **HS10** | **HS11** | **HS12** | **HS13** | **HS14** | **GE09** | **VK08** | **Total**  **Recapture** |
| --- | --- | --- | --- | --- | --- | --- | --- | --- | --- | --- |
| HS11/8085 | 0 | 1 | 9 | 124 | 8 | 13 | 0 | 5 | 0 | 160 |
| HS14/11804 | 0 | 2 | 16 | 76 | 21 | 21 | 0 | 2 | 10 | 148 |

**Table S2.**  Parameter estimates and 95% confidence intervals obtained for the model of mosquito movement using the Generalised-Poisson model for trap counts for the Green dust release.

| **Parameter** | **Maximum Likelihood Estimate** | **Lower 95% C.I.** | **Upper 95% C.I.** |
| --- | --- | --- | --- |
| Movement drift in x-direction ($\mu_{x}$) | -9.24 | -90.04 | 63.96 |
| Movement drift in y-direction ($\mu_{y}$) | -15.81 | -92.81 | 59.19 |
| Movement dispersion ($\sigma$) | 941.11 | 786.03 | 1149.41 |
| Death rate ($\beta$) | 0 | 0 | 2.78 E-3 |
| Wet season effect ($\alpha$) | -8.94 E-2 | -0.4799 | 0.4569 |
| Box fan trap catch rate modifier ($K_{BFT}$) | 4146.6 | 3133.9 | 5324.1 |
| Dispersion parameter of Generalised Poisson trap counts ($\varphi$) | 0.5616 | 0.3113 | 0.9447 |

**Table S3.**  Parameter estimates and 95% confidence intervals obtained for the model of mosquito movement using the Generalised-Poisson model for trap counts for the Orange dust release.

| **Parameter** | **Maximum Likelihood Estimate** | **Lower 95% C.I.** | **Upper 95% C.I.** |
| --- | --- | --- | --- |
| Movement drift in x-direction ($\mu_{x}$) | 357.59 | -792.33 | 75.67 |
| Movement drift in y-direction ($\mu_{y}$) | -5.71 | -821.37 | -399.37 |
| Movement dispersion ($\sigma$) | 1287.86 | 1067.95 | 1561.65 |
| Death rate ($\beta$) | 0 | 0 | 7.69 E-2 |
| Wet season effect ($\alpha$) | -0.5149 | -0.7008 | -0.1535 |
| Box fan trap catch rate modifier ($K_{BFT}$) | 11963 | 9505.8 | 14759.7 |
| Dispersion parameter of Generalised Poisson trap counts ($\varphi$) | 1.1959 | 0.0598 | 0.4165 |
